# A veracity dissemination consistency-based few-shot fake news detection framework by synergizing adversarial and contrastive self-supervised learning

**DOI:** 10.1038/s41598-024-70039-9

**Published:** 2024-08-22

**Authors:** Weiqiang Jin, Ningwei Wang, Tao Tao, Bohang Shi, Haixia Bi, Biao Zhao, Hao Wu, Haibin Duan, Guang Yang

**Affiliations:** 1 School of Information and Communications Engineering, Xi’an Jiaotong University, Xi’an, 710049 Shaanxi China; 2 School of Computer Science and Technology, Anhui University of Technology, Anhui, 243002 China; 3 China Academy of Electronics and Information Technology, China Electroncs Technology Group Corporation (CETC), Beijing, 100041 China; 4 Beihang University, Beijing, 100191 China; 5 Bioengineering Department and Imperial-X, Imperial College London, London, W12 7SL UK; 6 National Heart and Lung Institute, Imperial College London, London, SW7 2AZ UK; 7 Cardiovascular Research Centre, Royal Brompton Hospital, London, SW3 6NP UK; 8 School of Biomedical Engineering and Imaging Sciences, King’s College London, London, WC2R 2LS UK

**Keywords:** Few-shot fake news detection, LM-based pseudo prompt-tuning, Contrastive learning, Adversarial learning, News veracity dissemination consistency, Information technology, Electrical and electronic engineering, Computer science

## Abstract

With the rapid growth of social media, fake news (rumors) are rampant online, seriously endangering the health of mainstream social consciousness. Fake news detection (FEND), as a machine learning solution for automatically identifying fake news on Internet, is increasingly gaining the attentions of academic community and researchers. Recently, the mainstream FEND approaches relying on deep learning primarily involves fully supervised fine-tuning paradigms based on pre-trained language models (PLMs), relying on large annotated datasets. In many real scenarios, obtaining high-quality annotated corpora are time-consuming, expertise-required, labor-intensive, and expensive, which presents challenges in obtaining a competitive automatic rumor detection system. Therefore, developing and enhancing FEND towards data-scarce scenarios is becoming increasingly essential. In this work, inspired by the superiority of semi-/self- supervised learning, we propose a novel few-shot rumor detection framework based on semi-supervised adversarial learning and self-supervised contrastive learning, named Detection Yet See Few (DetectYSF). DetectYSF synergizes contrastive self-supervised learning and adversarial semi-supervised learning to achieve accurate and efficient FEND capabilities with limited supervised data. DetectYSF uses Transformer-based PLMs (e.g., BERT, RoBERTa) as its backbone and employs a Masked LM-based pseudo prompt learning paradigm for model tuning (prompt-tuning). Specifically, during DetectYSF training, the enhancement measures for DetectYSF are as follows: (1) We design a simple but efficient self-supervised contrastive learning strategy to optimize sentence-level semantic embedding representations obtained from PLMs; (2) We construct a Generation Adversarial Network (GAN), utilizing random noises and negative fake news samples as inputs, and employing Multi-Layer Perceptrons (MLPs) and an extra independent PLM encoder to generate abundant adversarial embeddings. Then, incorporated with the adversarial embeddings, we utilize semi-supervised adversarial learning to further optimize the output embeddings of DetectYSF during its prompt-tuning procedure. From the news veracity dissemination perspective, we found that the authenticity of the news shared by these collectives tends to remain consistent, either mostly genuine or predominantly fake, a theory we refer to as “news veracity dissemination consistency”. By employing an adjacent sub-graph feature aggregation algorithm, we infuse the authenticity characteristics from neighboring news nodes of the constructed veracity dissemination network during DetectYSF inference. It integrates the external supervisory signals from “news veracity dissemination consistency” to further refine the news authenticity detection results of PLM prompt-tuning, thereby enhancing the accuracy of fake news detection. Furthermore, extensive baseline comparisons and ablated experiments on three widely-used benchmarks demonstrate the effectiveness and superiority of DetectYSF for few-shot fake new detection under low-resource scenarios.

## Introduction

In recent years, the dissemination of fake news has become a significant concern due to its potential to undermine social stability and development^1,2^. Fake news can be defined as intentionally fabricated and disseminated false or misleading information aimed at deceiving audiences and potentially causing societal impacts. Fake news can be disseminated through various mediums, including social media, news websites, and even traditional media outlets. Fake news, often disseminated by manipulative actors with illegal intentions, aims to control public opinion, thereby serving their own agendas and gaining benefits pecuniary or ideologically. This manipulation, especially evident during presidential election campaigns, involves politicians controlling news, advertising, and propaganda. Furthermore, with the popularity of social media and the internet gradually becoming the mainstream channel for people to obtain information, fake news extremely likely to exacerbate societal divisions, erode trust, manipulate public opinion, lead to misleading behaviors, and damage the credibility of the media^3,4^.

In this regard, there have been numerous efforts and investigations aimed at reducing the harm caused by the rampant spread of fake news, including the development of advanced algorithms for detecting misinformation, implementing stricter regulations on social medias, and promoting public media literacy education^2,3^. Among them, FakE News Detection (FEND) is a promise also dynamic research field focused on devising and developing machine learning algorithms, techniques, and systems for automatic fake news textual classification^4^. Researchers in the FEND area aim to combat misinformation by developing sophisticated models that analyze and categorize content. The FEND models leverage a mix of natural deep learning (DL), language processing (NLP), and data mining technique to distinguish between credible news and false information, thus ultimately contributing to sustaining the overall integrity of information dissemination within society^1^. An appropriate clarifications about FEND is that, in the context of social media, it is primarily focused on identifying and classifying intentionally misleading news articles. Conceptually, FEND is regarded as rumor verification, a typical binary classification task that determines whether a Internet news article is a rumor by assigning it to predefined labels of Real or Fake^5–7^.

Until now, the prevailing methods for fake news detection (FEND) are fully supervised fine-tuning Pre-trained Language Models (PLMs), which have demonstrated impressive performance across a variety of linguistic understanding tasks^8^. These methods achieve rumor detection through the text classification models based on fine-tuning PLMs^5,9,10^, which involves adding extra classifiers on top of the PLMs and further training the models to meet the classification objectives. Despite of the success and superiority of these methods, the majority of them belong to fully supervised learning and follow the “*Train-from-Scratch*” learning paradigm, where the model optimization solely relies on the supervised training corpora^7^. As a result, these approaches often encounter generalization challenges when faced with conditions of label scarcity. Contemporary methods for FEND predominantly rely on extensive labeled datasets and incorporate labor-intensive information heavily. Hence, these traditional PLM fine-tuning based FEND methods exhibit sub-optimal performance in low-resource scenarios, falling short of ideal standards and performances. Moreover, the creation of high-quality supervised datasets is a process burdened by time and labor constraints, leading to significant research bottlenecks. To be specific, due to the dependency of downstream classifier for a sufficient number of training examples for parameter adjustment, applying this conventional fine-tuning paradigm in few-shot learning scenarios remains difficult and challenging.

In response to the aforementioned learning deficiency of conventional fully supervised PLM fine-tuning and the lack of annotated data for misinformation detection, there is an increasing trend towards fake news detection with minimal or no labeled data^7,10,11^. This shift towards zero-shot and few-shot learning methodologies in fake news detection aims to sustain robust model generalizability across a variety of real-world low-resource scenarios^4,5,10,12^. The study of researching FEND methodologies for low-resource scenarios learning is known as few-shot Fake News Detection. This aims to reduce the annotation costs of high-quality supervised datasets by utilizing a limited amount of labeled data. Simultaneously, it enables the model to learn domain-agnostic transferability, rapid adaptability (i.e., fast adaptation), and robust predictive performance^13^.

**Figure 1:**
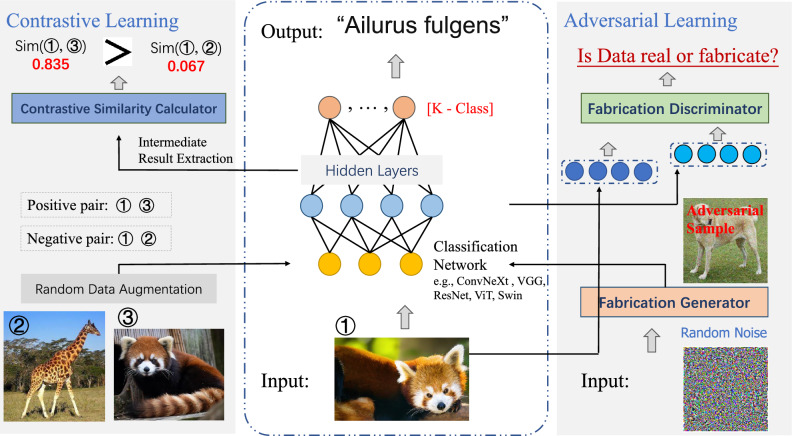
A typical conceptual diagram illustrating the paradigms of adversarial learning and contrastive learning within the context of an image classification task.

For deep learning solutions in data-scarce scenarios, there are currently two superior low-resource learning paradigms: contrastive learning^14–17^ and adversarial learning^18^, specifically as follows:

Contrastive learning: Inspired by human learning capabilities, contrastive learning has gained significant attention in the machine learning and AI communities for its ability to avoid extensive dataset labeling in self- and semi-supervised scenarios^7,10,11^. It contrasts features of different samples, using pseudo-labels as training signals to create enhanced representations for downstream tasks. This method is notably effective in computer vision and natural language processing, improving sentence-level semantic representations in low-resource settings^4,5,10,12^. The conceptual visualization of contrastive learning is shown in the left of Fig. 1.

Adversarial learning: Inspired by the system’s ability to learn and adapt in the face of adversaries, adversarial learning has revolutionized the ML and AI fields by enhancing model robustness and generalizability^14,16,17,19^. This approach, particularly effective in semi-supervised scenarios, leverages unlabeled data through adversarial processes to learn comprehensive representations. In addition to its contributions to computer vision (CV)^18,20^, contrastive learning has also made significant achievements in natural language processing (NLP) under data-scarce scenarios, particularly in enhancing sentence-level semantic representations through advanced representation contrastive techniques^16,17,21–23^. The conceptual visualization of adversarial learning is shown in the right of Fig. 1.

As mentioned above, contrastive learning and adversarial learning emerge as powerful paradigms within semi-/self-supervised learning, significantly advancing the development of artificial intelligence in low-resource scenarios. We are very interested in how contrastive and adversarial learning might improve few-shot FEND. Specifically, we focus on determining whether these methodologies can be helpful and to what extent they can enhance the fake news detection performance. Thus, in this work, we systematically investigates the potential of these two learning paradigms in the context of the few-shot FEND task. We primarily focuses on enhancing traditional PLM fine-tuning-based few-shot learning models by integrating contrastive and adversarial learning into the PLM-based prompt-tuning framework^7–9,24^, which efficiently boost the overall prediction performance, and generalizability of PLM-based models under low-resource FEND conditions.

Specifically, we develope a novel approach named “Detection Yet See Few” (DetectYSF), which is a veracity dissemination consistency-based few-shot fake news detection framework that synergizes adversarial and contrastive self-supervised learning. DetectYSF is built on Masked LM-based pseudo prompt learning^8–10,13^ and integrates a joint representation optimization mechanism during the model prompt-tuning stage, combining adversarial and contrastive learning. This allows it to achieve competitive performance even under conditions of sparse supervised data, with detection accuracy surpassing many current state-of-the-art (SOTA) baselines^6,11,25,26^.

Specifically, the proposed DetectYSF primarily includes the following novel features and modules, with the contributions of this paper presented as follows:

- Powerful few-shot capability of prompt learning: Firstly, our DetectYSF decisively moved away from the conventional fine-tuning technique, opting instead for prompt-based learning to directly elicit task-specific knowledge and harness pre-trained language models (PLMs) as powerful few-shot learners. Prompt-based learning^8^, also known as prompt tuning, is a constructed template-based learning paradigm that redefines fake news detection as a task-oriented text completion^7,10,13^ or text generation^9,24,27^ challenge, encapsulated within a natural language prompt. Among PLM-based methods, prompt-based learning has demonstrated significant promise in few-shot scenarios.
- Superiority of sentence representation contrastive learning: Subsequently, to optimize the prediction performance and robustness for DetectYSF, we employed a novel “*sentence representation-level contrastive learning*” strategy. This strategy involves constructing diverse sets of positive and negative contrastive sample pairs as DetectYSF undertakes few-shot learning. This process enables the model to discern and learn feature similarities and distinctions, thereby boosting its efficacy as a few-shot learner. Introducing contrastive learning in resource-scarce environments parallels the application of Meta Learning^23,28^ in the domain of supervised learning, specifically tailored for the FEND task. This strategic application aids the PLM encoder in producing more meaningful and robust sentence representations, thereby enhancing the learning and generalization abilities of prompt-based learning.
- Excellence of sample-level adversarial learning: To further enhance the output representation, generalization, and robustness of DetectYSF in rumor classification, we incorporated a novel “*training sample-level adversarial learning*” technique. This technique, based on the Generative Adversarial Network (GAN) framework, utilizes two types of generators and one discriminator: the Negative Sequence & Language Encoder Driven Engine (NegSeq-LMEncoder Engine), the Noise & Multilayer Perceptron (MLP) Driven Engine (Noise-MLP Engine), and the Binary Classification Contrast Learning Discriminator (BinCon Discriminator). This “*training sample-level adversarial learning*”, not only strengthens the DetectYSF’s resilience against deceptive inputs but also fine-tunes its capability to generalize from limited training examples, solidifying its rumor predictive performance across varied social contexts.
- Adoption of social context-based veracity dissemination consistency: To leverage social dynamics in verifying news authenticity, DetectYSF assesses the consistency and reliability of news based on social context clues by leveraging the “news veracity dissemination consistency” theory or phenomenon. This theory suggests that social users who frequently share the same news typically reflect broader social sentiments, marked by specific cognitive biases. The authenticity of the news shared by these collectives tends to remain consistent, either mostly genuine or predominantly fake. By integrating this understanding, DetectYSF enhances its predictive accuracy. Specifically, during post-processing, we introduced a novel “veracity feature fusion” strategy involving the construction of neighborhood dissemination sub-graphs. This strategy retrieves surrounding news nodes and integrates them through veracity feature transfer, refining and optimizing the original prompt-tuning based predictions. This approach not only improves detection accuracy but also provides deeper insights into the social mechanisms influencing news circulation.

In summary, we introduce the powerful framework: Detection Yet See Few (DetectYSF) for few-shot fake news detection in this paper. Extensive comparative and ablation experiments on three widely-used datasets (PolitiFact, GossipCop, FANG) show that DetectYSF surpasses many state-of-the-art baselines in terms of accuracy, including PET^11^, KPT^9^, and Prompt-and-Align^7^, verifying its effectiveness in few-shot FEND. For instance, DetectYSF achieved an accuracy of 83.94% on GossipCop dataset, compared to 62.97% by PET and 62.19% by KPT. Practically, DetectYSF can serve as a powerful engine for rumor detection in various real-world applications, contributing positively to societal health and development. To support further research, our code is available on GitHub: https://github.com/albert-jin/Self-Supervised-fewshot-FEND-Enhance.

The remainder of this paper is structured as follows: The “Related work” section provides an extensive studies review, focusing on prior methods for fake news detection, with recent advancements in contrastive and adversarial learning techniques. The “Methodology preliminaries” section introduces the foundational concepts related to few-shot fake news detection, including the definitions of news veracity dissemination consistency. The “Detection yet see few” section details the architecture of our novel Detection Yet See Few (DetectYSF) framework. The “Experiments” section presents a series of experiments, including main results and ablated studies, to demonstrate the efficacy of DetectYSF against several baselines in few-shot scenarios. Finally, The “Conclusion” section provides a summary of the key contributions of our research and discusses potential avenues for future development and enhancement of fake news detection frameworks.

## Related work

### Few-shot fake news detection

Fake news detection (FEND) is not simple but rather is a complicated problem, which requires multiple processes to recoginze the authenticity category of a given news article (textual document)^2,3^. Apart from various conventional feature engineering-based machine learning (ML) methods, such as naïve Bayes, decision tree, logistic regression, KNN, and SVM, numerous NLP researchers have introduced and implemented various competitive deep learning (DL)-based techniques^5,6,26,29^.

Sheng et al.^12^ expanded their focus from observing language patterns (“zoom in” mode) to considering the broader external news environment where fake news spreads (“zoom out” mode). They introduced the News Environment Perception Framework (NEP) to account for both macro and micro news environments. Wu et al.^7^ developed Prompt-and-Align, a novel few-shot fake news detection method based on prompt-tuning, which leverages both pre-trained knowledge in PLMs and external knowledge from social context topology. They introduced a news proximity graph, represented by a graph adjacency matrix, to capture veracity-consistent signals in shared readerships. Nan et al.^6^ redirected their research towards interdisciplinary fake news detection. They proposed a Multi-Domain Fake News Detection method (MDFEND) and released the Chinese multi-domain fake news detection dataset “Weibo21”. Lee et al.^10^ used perplexity scores from evidence-conditioned language models to estimate the credibility of claims and their corresponding evidence. The method identifies the optimal threshold to distinguish between “Supported” and “Unsupported” claims on a validation set. Wang et al.^30^ proposed the Event Adversarial Neural Network (EANN), an end-to-end framework designed to address the challenge of detecting fake news on newly emerged events by extracting event-invariant features while neglecting domain-specific features. Silver et al.^31^ introduced a framework that jointly preserves both domain-specific and cross-domain knowledge, enhancing fake news detection across various domains. They also developed an unsupervised technique for selecting informative unlabelled news records for manual labelling. Petrou et al.^32,33^ proposed a multiple change-point detection framework aimed at analyzing the linguistic characteristics of news articles to identify and track changes in the linguistic features of real and fake news over time. Maham et al.^29^ proposed an end-to-end framework named Adversarial News Net (ANN) for robust fake news classification using adversarial training. By extracting emoticons and integrating them into the model, they improved the performance of the classification. Hu et al.^34^ investigated the potential of large language models (LLMs) in fake news detection, finding that while sophisticated LLMs like GPT-3.5 can expose fake news and provide multi-perspective rationales, they still underperform compared to fine-tuned small language models (SLMs) like BERT.

Overall, these studies have collectively advanced the FEND field by integrating diverse methodologies and innovative frameworks^35–38^. The ongoing DL-based technique development has significantly been beneficial to identify and mitigate the impact of misinformation across various social contexts and everyday life scenarios.

### Contrastive Learning

Inspired by the rapid learning capabilities of humans, contrastive learning is recently proposed and gradually receives much attention from the machine learning (ML) and artificial intelligence (AI) community^14,16,17,19^. Contrastive learning, a typical and effective approach within self-supervised paradigms, circumvents the need for extensive dataset labeling, demonstrating its superiority in self-/semi- supervised research fields. Specifically, it utilizes contrasts between features of different samples, treating custom pseudo-labels as training signals. This makes it an outstanding enhancement method for constructing meaningful representations and training model in low-resource learning scenarios, particularly proven effective in various AI applications.

Contrastive learning has also made significant achievements in natural language processing (NLP) under data-scarce scenarios, particularly in enhancing sentence-level semantic representations through advanced representation contrastive techniques^16,17,21–23^. Xu et al. provides a comprehensive review, and systematically summarizes and categorizes the contrastive learning based sentence representation models. Gao et al. proposed SimCSE^16^, a simple contrastive learning framework for sentence embeddings, which leverages dropout as minimal data augmentation in an unsupervised approach and incorporates natural language inference datasets for a supervised approach. Inspired by deep metric learning (DML), Giorgi et al. propose DeCLUTR^25^, a self-supervised model designed to learn universal sentence embeddings without the need for labeled data. Kim et al. propose NT-Xent^23^, a self-guided contrastive learning method to enhance the quality of BERT sentence representations. The NT-Xent training objective for sentence representation learning fine-tunes BERT in a self-supervised manner without relying on data augmentation. Wu et al. proposed CLEAR^22^, a contrastive learning framework for sentence representation that employs multiple sentence-level augmentation strategies to learn noise-invariant sentence representations. Yan et al. proposed ConSERT^17^, a contrastive learning framework for self-supervised sentence representation transfer, which fine-tunes BERT to address the collapse issue in sentence representations.

### Adversarial learning

Adversarial training is a critical approach to increasing the robustness of neural networks^18,39^, which has proven particularly effective in semi-supervised learning scenarios where labeled data are scarce. In this process, samples are augmented with slight perturbations (small changes that are likely to cause misclassification), and the neural network is trained to adapt to these changes, thus becoming more resilient to adversarial examples. Adversarial learning introduces a dynamic where models learn to predict not only from genuine data but also from artificially generated adversarial examples. Unlike contrastive learning, the essence of adversarial learning resides in the adversarial examples construction and the strategies for learning from these adversarial samples. By leveraging unlabeled data through adversarial processes, models can learn more comprehensive representations, enhancing their applicability in domains such as computer vision (CV)^18,20^, natural language processing (NLP)^29,39–42^, and graph representation learning^28^.

In semi-supervised adversarial training, a notable example is Generative Adversarial Networks (GANs)^20^. GANs serve as a prominent adversarial training framework, aiming to develop models that can either withstand or capitalize on adversarial conditions, thus enhancing model performance. GANs are composed of a generator that produces samples designed to replicate the real data distribution, and a discriminator that endeavors to differentiate between real and generated data.

Alsmadi et al.^18^ discusses the increasing importance of adversarial machine learning (AML) in the context of machine learning algorithms (MLAs), highlighting their susceptibility to adversarial attacks designed to manipulate model outputs. Wang et al.^39^ introduces the foundations of deep adversarial learning in NLP, focusing on the challenges and advancements of applying models like GANs to linguistic tasks. It categorizes different types of adversarial learning models, including adversarial examples, training, and generation, and discusses their applications and future directions in NLP. Miyato et al.^40^ propose adversarial and virtual adversarial training methods for semi-supervised text classification by applying perturbations to word embeddings in recurrent neural networks. Croce et al. introduces GAN-BERT^41^, a model that combines BERT with Semi-Supervised Generative Adversarial Networks (SS-GANs) to improve text classification with minimal labeled data. Mei et al. presents CON-GAN-BERT^42^, which combines contrastive learning, GAN, and BERT for sentence-level sentiment classification, achieving superior performance on few-shot learning tasks without data augmentation or unlabeled data. Wu et al. introduces GACN^28^, a novel graph neural network that combines Generative Adversarial Networks (GANs) with Graph Contrastive Learning (GCL) to improve graph representation learning.

## Methodology preliminaries

### Defintion of fake news detection

Given a particular news article item $a$, which is a series of textual argument descriptions towards a specific event or a viewpoint, the goal of fake news detection is to determine the authenticity of $a$, operationalized by a function $F$, which maps $a$ to a binary outcome $\{0, 1\}$. Given the above defined notations and prerequisites, FEND can be formulated as:

$$\begin{aligned} F(a) = {\left\{ \begin{array}{ll} 1, &{} \text {if a is identified as fake, misleading},\\ 0, &{} \text {if a is verified as true, trustworthy}. \end{array}\right. } \end{aligned}$$

Here, $F$ denotes the predictive algorithm intended to be learned and modelled. Fake news detection is treated as a binary classification problem because fake news essentially distorts information due to manipulation by its publisher. Such misinformation is deliberately crafted to mislead the audience, often reflecting the publisher’s intent to control narratives and influence opinions, thus requiring effective detection mechanisms to accurately classify it.

Moreover, considering a set of user dissemination engagement records $E$ involving $n$ users centred around the social contexts of the aforementioned news item $a$, we can construct social context-based news dissemination network $N$. Alongside the news $a$, together with the reposting records $E$ involving $n$ users and the constructed network $N$, the fake news detection task can be further formulated as:

$$\begin{aligned} F(a, E, N) = {\left\{ \begin{array}{ll} 1, &{} \text {if a is identified as fake, misleading},\\ 0, &{} \text {if a is verified as true, trustworthy}. \end{array}\right. } \end{aligned}$$

Given: the news item $a$, the dissemination engagement records $E$ of $a$ and the constructed social context-based news dissemination network $N$ surrounding the social contexts of $a$;

Target: predicting the correct veracity categories $l$ from Real or Fake for the news item $a$.

It is worth noting that: Although the conceptual modeling for FEND can be represented by the simple formulation mentioned above, the proposed FEND methodologies may encompass a series of complex steps, starting with numerous preprocessing or data mining techniques. Therefore, the technical difficulty of exploring FEND will become increasingly important in the future, along with its potential applications.

### News veracity dissemination consistency

Due to the psychological tendencies and cognitive traits of Internet social users, these individuals often gravitate towards, absorb, and trust information that aligns with their ideological views^1,2,7^. The authenticity of the news shared by these collectives tends to remain consistent, either mostly genuine or predominantly fake. This leads to scenarios where erroneous content is perceived and shared as factual within groups of like-minded individuals, a theory or phenomenon we define as the “news veracity dissemination consistency”. Specifically, the phenomenon can be concluded as: Social users who frequently share the same news typically reflect broader social sentiments, marked by specific cognitive biases. Additionally, the truthfulness of the news circulated by these groups usually stays uniform, being either largely authentic or primarily false.

To elaborate further, Naive realism (the tendency of individuals to believe information that aligns with their perspectives more easily), confirmation bias (i.e., confirmation bias^43^, the inclination to seek and favor information that supports pre-existing beliefs), and normative influence theory (the choice to share and consume options deemed socially acceptable for approval and validation) are commonly considered key influences in the fake news recognition and dissemination about the “news veracity dissemination consistency” theory^1^.

Social homophily^1^—where social users tend to establish connections with others who share similar ideologies, and personalized recommendation algorithms— recommending connections with similar social users for friendship or follow. Additionally, this biased social behavior, motivated by personal cognition, positions, and beliefs, far surpasses verification against objective facts. These factors contribute to the formation of echo chambers and filter bubbles, thereby exacerbating the prevalence of “news veracity dissemination consistency”.

Shu et al. analyze the psychological factors influencing the dissemination of fake news through the lens of confirmation bias. Sharma et al. examine the existence and spread of fake news from both sociological and psychological perspectives, focusing on how confirmation bias affects individual and societal behavior.

The exacerbation of political polarization^44^, particularly in the United States, is a typical example of “news veracity dissemination consistency” influenced by the fragmentation of news media and the dissemination of misleading information on social media. Researchers found that social users with similar ideologies in politics tend to cluster and engage more with allies, while distancing themselves from opposing forces. In other words, individuals with similar political ideologies tend to cluster together, sharing news that aligns with their beliefs, which can be either predominantly genuine or mostly fake.

From the viewpoint of the FEND task^7^, the relationship between the collection of news articles a user interacts with and the consistency of their veracity signals is essential to FEND. This is particularly vital in few-shot scenarios, where external knowledge plays a significant role in helping to verify whether a given news article is rumor or not.

## Detection yet see few (DetectYSF)

This section mainly introduces the architecture, main components, and operation of the Detection Yet See Few (DetectYSF) framework.

### Masked LM-based prompt learning for rumor classification

Given the advantages of prompt-tuning over conventional fine-tuning in learning under data-scarce scenarios (few-shot learning), DetectYSF employs a “Masked Language Modeling (MLM)-based pseudo prompt learning” paradigm on top of Transformer-based PLMs (e.g., BERT, RoBERTa^7,45^) for the primary network architecture in rumor classification.

As illustrated in “Introduction”, MLM-based pseudo prompt learning^10,13,46^ leverages the pre-training objective of BERT series models, specifically the MLM task, to reframe fake news detection as a masked token prediction problem. By constructing a prompting template, DetectYSF transforms the original pre-training MLM task from PLMs into a “News Veracity-oriented LM” task, which effectively extracts task-specific knowledge from the PLM, significantly reducing the need for a large supervised dataset.

Firstly, rumor classification aims c *K* news articles $\mathscr {A} = {a_{0}, a_{1}, \ldots , a_{K}}$ into a class label $y \in \mathscr {Y}$, which is also the mathematical formulation used in traditional PLM fine-tuning. In contrast, prompt Learning reformulates classification into a [MASK] blank-fill question with specific pre-defined label word set $\mathscr {Z}$. Instead of directly using $\mathscr {A}$, it searches for an appropriate label word $z \in \mathscr {Z}$ based on the prompt $f_{fill}(\mathscr {A})$, where $f_{fill}(\cdot )$ is the prompting function (template).

Let $\mathscr {M}$ be the adopted Transformer-based PLM backbone (i.e., BERT, RoBERTa^7,45^). Given a textual input, $\mathscr {M}$ provides the probability distribution of vocabulary words for each token position in the input textual sequence, thus faithfully simulating the MLM task during its pre-training stage. Given a news article $a_{i} \in \mathscr {A}$, we manually construct a simple yet explicit prompt template *T* as follows: T($\cdot$): “It is [MASK] that [SLOT of $a_{i}$].”. The template *T* includes a slot for the special [MASK] token and another for news article $a_{i}$. These are combined with the news input $a_{i}$ to form the prompt fed to PLM, which can be formulated as Eq. (1).

1 $$\begin{aligned} \textbf{T}(\mathbf {a_{i}}) = It \hspace{5.0pt}is \hspace{5.0pt}[MASK] \hspace{5.0pt}that \hspace{5.0pt}\mathbf {a_{i}}. \end{aligned}$$

Taking $\textbf{T}(\mathbf {a_{i}})$ as $\mathscr {M}$ input, $\mathscr {M}$ outputs a score logits distribution of vocabulary words for each token in the textual input, represented by $\mathbb {V}$. Here, $v_{i} \in \mathbb {R}^{|\mathscr {V}|}$ and $\mathscr {V}$ denotes the length of the PLM vocabulary, as depicted in Eq. (2).

2 $$\begin{aligned} \mathscr {V} = \mathscr {M}(\textbf{T}(\mathbf {a_{i}})) =[v_{[CLS]}, v_{[It]},...,v_{[MASK]},...,v_{[SEP]}]. \end{aligned}$$

**Figure 2:**
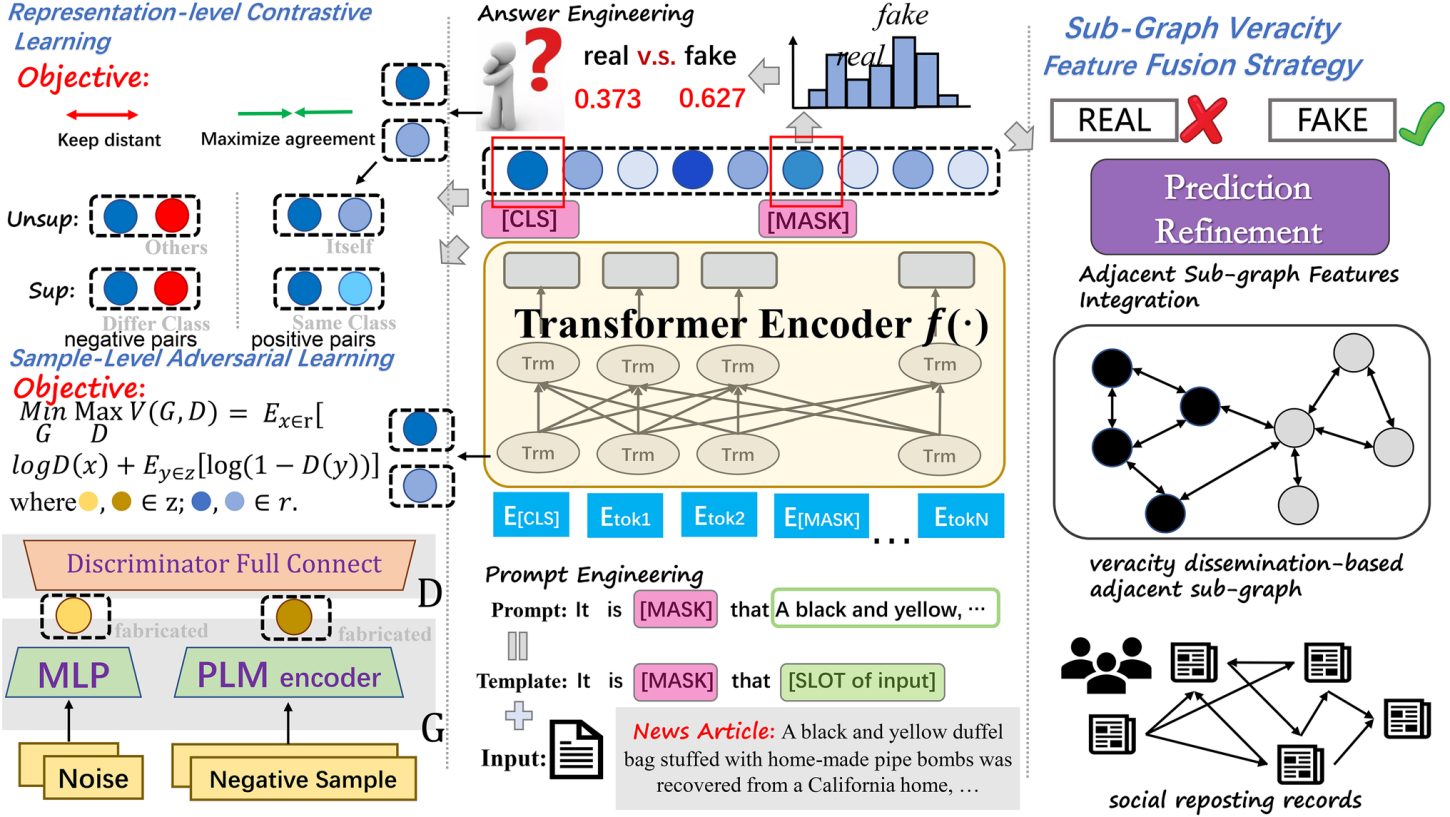
The model architecture of our proposed DetectYSF, which is a MLM-based prompt learning framework for rumor classification (center). Utilizing the *Sentence Representation Contrastive Learning* (top-left) and *Sample-Level Adversarial Learning* (bottom-left) to further enhance the few-shot capability for the prompt-tuning based classification task. The *Social Context-based Veracity Dissemination Consistency* is then considered via the “veracity feature fusion” strategy during inference stage (right).

Afterwards, we consider optimizing the vocabulary score logits distribution $v_{[MASK]}$ at the token position of [MASK]. At inference, the decoding process for the logits vector $v_{[MASK]}$ is closely associated with the *Answer Mapping Engineering* in prompt learning^8^. We then define the label word set $\hat{Z}$ as $\mathscr {Z} = \{``real'', ``fake''\}$ to use in our fake news detection task involving [MASK] token filling. Given $\hat{Z}$, we retrieve and extract the conditional probability logit $Prob_{z}(v_{[MASK]})$ of each label word *z* (where $z \in \hat{Z}$) from the score logits distribution $v_{[MASK]}$. Let *y* being the label corresponding to label word *z* in label space $\mathscr {Y}$, $y \in \mathscr {Y}$ for FEND. Ultimately, the probability score for each label word *z* is computed using the softmax function based on score logits $v_{[MASK]}$, as illustrated in Eq. (3).

3 $$\begin{aligned} P_{y,z}(v_{[MASK]})= Argmax_{v_{[MASK]}}(y,z) = \frac{\exp \left( Prob_{z}(v_{[MASK]})\right) }{\sum _{\hat{z} \in \mathscr {Z}} \exp \left( Prob_{\hat{z}}(v_{[MASK]})\right) }, \qquad where \quad z \in \mathscr {Z} \quad maps \quad to \quad y \in \mathscr {Y}. \end{aligned}$$

Then, we utilize the probability score $P_{y,z}(v_{[MASK]})$ of the token at [MASK] position to determine the most appropriate label word (answer) *y*, and then leverage the pre-defined answer mapping function, i.e., “real” $\rightarrow$ 0 (real) and “fake” $\rightarrow$ 1 (fake), to map the answer *y* to the target category in $\mathscr {Y}$.

Overall, it stands in stark contrast to traditional PLM fine-tuning. Pseudo prompt-tuning based on MLM fully leverages the extensive linguistic knowledge embedded in PLMs, yielding competitive performance compared to traditional PLM fine-tuning methods.

### Sentence representation contrastive learning strategy

Contrastive learning can significantly enhance the overall robustness and convergence capability of various supervised models in data-scarce scenarios. Building on previous contrastive learning strategies for optimizing LM representation vectors, we employed a novel “*sentence representation-level contrastive learning*” strategy in DetectYSF.

Since the core idea of contrastive learning is to bring the similarity of positive pairs closer while pushing the similarity of negative samples further apart, defining and collecting positive and negative sample pairs becomes a crucial aspect of research. In this work, the design of these contrastive sample pairs draws inspiration from the Unsupervised and Supervised methodologies of Simple Contrastive Sentence Embeddings (SimCSE)^16,17,22^, as shown in the top-left of Fig. 2, in which the following strategies were introduced:

1. Unsupervised Contrastive Sample, where representation outputs from the same news article (including the article itself) at different instances via a PLM encoder are considered positive contrastive pairs, and representation outputs from different articles are negative pairs.

Specifically, these unsupervised approach is inspired by the practice in visual representation of applying random transformations to the same image (e.g., word deletion, reordering, and substitution)^14,19^. This unsupervised contrastive samples are constructed through the use of standard dropout masks operations on a Transformer-based LM encoder, while avoiding random augmentation techniques such as word cutoff, token shuffling, and substitution^16,17,22,25^.

1. Unsupervised positive contrastive pairs sampling: Due to the presence of standard dropout (default p = 0.1 in BERT and RoBERTa) between the Multi-Head Self-Attention, fully connected layers, and Add & Norm layers within the Transformer-based LM block, it can introduce random perturbations to the LM’s final output. Therefore, passing the same language textual sequence through the same Transformer-based LM encoder twice yields vectors with different values but consistent semantics, making them can serve as natural and appropriate positive contrastive pair samples.
2. Unsupervised negative contrastive pairs sampling: For the selection of negative contrastive samples, we directly take sentences different from the current news article input from the supervised data, pairing them as a set of negative sample inputs, and then obtain the negative contrastive representation pairs through the LM encoder.

For simplicity, we provide a formal description of the unsupervised collective approach. First, we treat all news articles from the supervised dataset (excluding their labels) as the data source for unsupervised contrastive learning. Assuming this data source includes $N$ sentences $S = \{s_{i}\}_{i=1}^{N}$, we randomly select $s_{i}$ from $S$ as the original news article. We define the positive sample pair as $\{s_{i}, s_{i}^{+}\}$, where $s_{i}^{+} = s_{i}$, and the negative sample pair as $\{s_{i}, s_{i}^{-}\}$, where $s_{i}^{-} \in S$ and $s_{i}^{-} \ne s_{i}$. In the specific experimental implementation, during each training iteration, we only select a fixed number $K$ (default 8) of $s_{i}^{-}$ from $S$ and combine them with the positive sample pair $\{s_{i}, s_{i}^{+}\}$ to form a learning batch (*i.e.,* $\{s_{i}, s_{i}^{+}, s_{i1}^{-}, \ldots , s_{iK}^{-}\}$) for joint optimization.

2. Supervised Contrastive Sample, where representation outputs from news articles within the same veracity category (including the article itself) are positive pairs, *i.e.,* Fake & Fake and Real & Real, and representation outputs of articles from different veracity categories are negative pairs, *i.e.,* Fake & Real.

Specifically, we explore the potential of FEND supervision signals, i.e., the annotated news veracity labels: *real/fake*, to contrast features between news articles, further enhancingthe generalization ability of DetectYSF in few-shot scenarios. Compared to previous textual data augmentation methods, we ignore various data augmentation strategies such as random cropping and synonym replacement in this process. Instead, we directly adopt original sentences with the same FEND label as positive sample pairs, and select original sentences with different FEND labels as negative sample pairs.

For ease of understanding, a simple and intuitive formalization of the supervised contrastive approach is as follows:

First, we treat all news articles from the supervised dataset with their veracity labels as the data source for supervised contrastive learning. Assuming this data source includes $N$ sentences $S = \{s_{i}\}_{i=1}^{N}$, we randomly select $s_{i}$ from $S$ as the original news article.

1. Supervised positive contrastive pairs sampling: We define the positive sample pair as $\{s_{i}, s_{i}^{+}\}$, where $s_{i}^{+}$ is another sentence from $S$ with the same veracity label as $s_{i}$.
2. Supervised negative contrastive pairs sampling: The negative sample pair is defined as $\{s_{i}, s_{i}^{-}\}$, where $s_{i}^{-}$ is a sentence from $S$ with a different veracity label from $s_{i}$.

Similarly, to maintain consistency with the unsupervised ones, we also set a fixed number $K$ of $s_{i}^{-}$ from negative samples (default 8), and combine these $K$ negative samples with the initial positive sample pair $\{s_{i}, s_{i}^{+}\}$ to form a learning batch (*i.e.,* $\{s_{i}, s_{i}^{+}, s_{i1}^{-}, \ldots , s_{iK}^{-}\}$) for joint optimization.

3. Un-/Supervised Contrastive Learning Objective Optimization The optimization objective function for both unsupervised and supervised contrastive approach is defined as Eq. (4). This objective function is gradually minimized during training. During DetectYSF’s few-shot training, the mini-batch size is typically set as $K+1$, where *K* represents the number of negative samples, and 1 represents the count of positive samples.

4 $$\begin{aligned} \mathscr {L}_{contrast}^{si} = - \log \frac{\exp (Sim(s_i, s_i^+) / \tau )}{\exp (Sim(s_i, s_i^+) / \tau ) + \sum _{j=1}^{K} \exp (Sim(s_i, s_j^{-}) / \tau )}. \quad where\quad Sim(x, y) = Cosine (x, y) = \frac{x \cdot y}{\Vert x\Vert \Vert y\Vert } \end{aligned}$$

Here, the pairwise similarity between representation embeddings is calculated using cosine similarity, i.e., $\text {Sim}(\cdot ) = \frac{x \cdot y}{\Vert x\Vert \Vert y\Vert }$; The temperature coefficient $\tau$ adjusts the scale of the distance function output, thereby affecting the smoothness of the softmax function and its sensitivity to different similarities. Following the SimCSE setup^16^, we select the optimal configuration $\tau = 0.05$.

It’s worth noting that in Transformer-based LMs such as BERT and RoBERTa, the ‘[CLS]’ token’s output vector, after being processed by multiple layers of Transformer encoders, is considered a condensed representation of the global semantic information of the entire input sequence. This output vector is commonly used for subsequent sentence-level feature classification. Therefore, as shown in the center of Fig. 2, we use the embedding output of the ‘[CLS]’ token position as the intermediate representation for the contrastive learning optimization of DetectYSF.

### Sample-level adversarial learning strategy

As we all know, the performance of any deep learning (DL) neural network model relies heavily on high-quality training sets. However, in data-scarce training settings, few-shot DL models struggle to learn from adversarial noise without additional external supervised signals. Meanwhile, adversarial machine learning (AML)^18^, acting as a special semi-supervised paradigm, addresses this issue by incorporating adversarial examples into the training process, which helps DL models learn adversarial samples and enhance prediction accuracy.

As shown in the bottom-left of Fig. 2, our DetectYSF leverages AML principles by introducing a novel “*sample-level adversarial learning*” strategy. This strategy boosts the model’s resistance to knowledge bias caused by noise, significantly improving generalization and robustness. Specifically, following the foundational setup of the Generative Adversarial Network (GAN)^18,20^, DetectYSF incorporates two types of generator modules and an innovative discriminator module:

1. Negative Sequence & Language Encoder Driven Engine (NegSeq-LMEncoder Engine): This generator is a LM encoder-driven generation engine that inputs random sequences of words from the vocabulary, selections of news articles with labels different from the current input, and semantically altered versions of the input news by adding negating terms.

NegSeq-LMEncoder Engine uses an independent LM encoder which differs from the backbone of DetectYSF to keep its parameter optimization independent. Inspired by noise generation methods in GANs, this engine heuristically simulates noise generation. Due to the textual sequence input for the LM encoder, we adopt the following two sampling strategies:

1. Noise sampling based on random word construction: First, calculate the length distribution of all news articles in the training and test sets and the word frequency. Then, construct fabricated news sentences that follow the aforementioned word frequency distribution and sentence length distribution.
2. Noise sampling based on negative sample text augmentation: First, sample negative examples that do not match the current sentence’s veracity label from the training samples. Then, perform textual augmentation on these negative samples with a certain probability, including a series of word-level random perturbations such as random token cropping, token swapping, and synonym replacement.

Following DetectYSF, by inputting the aforementioned noise sentences into the NegSeq-LMEncoder Engine’s Transformer encoder and obtaining the fabricated representation embeddings at the ‘[CLS]’ token position after decoding. For subsequent formulated modeling, we denote these embeddings as $\hat{E}_{plm}$, and this LM-based discriminator, NegSeq-LMEncoder Engine, is denoted as $G_{plm}$.

2. Noise & Multilayer Perceptron (MLP) Driven Engine (Noise-MLP Engine): This generator is driven by a multilayer perceptron (MLP) and inputs random noise to create textural perturbations.

In addition to the LM encoder-based fabricated representation generator, we have introduced a generator based on a Multilayer Perceptron (MLP), called the Noise & Multilayer Perceptron (MLP) Driven Engine (Noise-MLP Engine). This simple network, following the initial settings of GAN-BERT^41^, uses multiple fully connected layers. The input to the network is a 100-dimensional noise vector drawn from $N(\mu , \sigma ^{2})$. The network consists of two layers, with an internal dimension of 512 and an output dimension of 768, matching the hidden layer dimensions of most LMs. LeakyReLU is used as the activation function, and the dropout rate for the intermediate layer is set at 0.1.

To ensure consistency in the subsequent discriminator’s identification process, the fabricated embeddings generated by the Noise-MLP Engine must match the dimensions of the embeddings produced by DetectYSF (i.e., the dimension is 768 for BERT and RoBERTa). For the ensuing formal modeling, we refer to these embeddings as $\hat{E}{mlp}$, and designate this MLP-based discriminator, Noise-MLP Engine, as *G* *mlp*.

3. Binary Classification Contrast Learning Discriminator (BinCon Discriminator): This novel discriminator module, inspired by the original GAN discriminator^20,40,47^, is designed to classify whether the representations are genuine or fabricated. It evaluates the unmodified representations produced by the LM encoder (original) and the adversarial representations generated by the aforementioned generators (fabricated).

Specifically, the BinCon discriminator is an MLP classifier designed to identify whether a representation embedding is genuine or fabricated. It takes representation vectors $E^{*}$ from the original DetectYSF, NegSeq-LMEncoder Engine, and Noise-MLP Engine, where $E^{*} = \{E, \hat{E}_{plm}, \hat{E}_{mlp}\}$. Here, $E^{*}$ can either be the genuine representation *E* from the real news article distribution or the fabricated embeddings $\hat{E}_{plm}$ and $\hat{E}_{mlp}$ produced by the generators $G_{plm}$ and $G_{mlp}$. The BinCon discriminator outputs a 2-dimensional logits vector that corresponds to the genuine and fabricated categories.

4. Adversarial learning objective optimization. Formally, given the embedding obtained at the ‘[CLS]’ token position from the original news article input into DetectYSF (genuine representations); the fabricated representations produced by the aforementioned generators; the generators, and the discriminator, the adversarial optimization objective of “*sample-level adversarial learning*” strategy can be formulated as follows (Eqs. 5 and 6).

5 $$\begin{aligned} L_{adver} = \min _{G} \max _{D} V(D, G) = \mathbb {E}_{E \sim p_{\text {data}}(E)} [\log D(E)] + \mathbb {E}_{\hat{E} \sim p_{G}(\hat{E})} [\log (1 - D(\hat{E}))]; \end{aligned}$$

6 $$\begin{aligned}&where \quad E = DetectYSF(p_{\text {data}}), \quad \hat{E} = G(p_{G}). \end{aligned}$$

Here, *E* represents the genuine embeddings obtained from the original news article input; $\hat{E}$ represents the fabricated embeddings produced by the generators $G_{plm}$ and $G_{mlp}$; *G* denotes the generators $G_{plm}$ and $G_{mlp}$; *D* denotes the discriminator; $p_{\text {data}}(E)$ is the real distribution of the real data; $p_{G}(\hat{E})$ is the fabricated distribution of the generated data.

During the model training process, we asynchronously and in parallel optimize both the discriminator (*D*) and the generators (*G*). For the discriminator, the goal is to train it to maximize the probability of correctly classifying the representation as genuine or fabricated, thereby enhancing its ability to distinguish real features from fake noise. For the generators, the objective is to optimize them so that the fabricated samples produced from random noise are indistinguishable by the discriminator. This establishes a dynamic interplay between the discriminator *D* and the generators *G*, converging to an optimal Nash equilibrium state.

Additionally, inspired by the regularization constraint on vector similarity considered in GAN-BERT^41^, we also introduced a “*feature matching*” objective loss, as specified in Eq. (7). This optimization objective takes into account that the generator should produce examples whose intermediate representations, when provided as input to the discriminator, are very similar to the real ones (measured using the Euclidean L2 norm distance $||\cdot ||_{2}^{2}$).

7 $$\begin{aligned} L_{feature\_matching} = ||E - \hat{E}_{plm}||_{2}^{2} + ||E -\hat{E}_{mlp}||_{2}^{2} \end{aligned}$$

Finally, the total loss function for adversarial learning is defined as the weighted sum of the aforementioned sub-losses, $L_{adver}, L_{feature\_matching}$, controlled by a hyper-parameter $\delta$ ranging between 0 and 1 (default 0.5), as specified in Equation 8. This objective function is gradually minimized during training.

8 $$\begin{aligned} L_{total} = \delta L_{adver} + (1- \delta ) L_{feature\_matching} \end{aligned}$$

Overall, the adversarial learning strategy focuses on enhancing the authenticity recognition ability of these fabricated representations apart from real FEND samples. By using the fabricated embeddings generated by the generators, it enhances the BinCon discriminator’s ability to recognize authenticity. This joint training process provides the LM encoder in the DetectYSF primary network with guiding biases, thereby improving the robustness and generalization of the DetectYSF framework for detecting fake news under few-shot conditions.

### Neighborhood dissemination sub-graph veracity feature fusion

There exists a predominant psychological phenomenon called “*Confirmation Bias*”^1,2,43^: People typically seek information that reinforces their preferred hypotheses or current beliefs and tend to interpret this information in a way that favors these hypotheses or beliefs. On the other hand, they usually avoid seeking or even ignore information that contradicts their hypotheses or beliefs and that might support alternative perspectives.

By examining the characteristics of how fake news spreads, we developed a theory called “*news veracity dissemination consistency*”: The truthfulness of news circulated by these groups tends to remain consistent, either being largely authentic or primarily false. This social context-based veracity signal from the phenomenon is crucial for FEND task, aiding in determining whether a given news article is a rumor or not. It is worth noting that in real life, there are always social groups composed of people or things with similar or consistent views, as the Chinese proverb says: Ren Yi Lei Ju, Wu Yi Qun Fen, i.e., “Birds of a feather flock together.”.

Therefore, we consider using representative similarities such as social users relationship, users’ interest preference, and news reposting pattern to assist our DetectYSF in optimizing the veracity/authenticity prediction of news article disseminated by users.

Specifically, during the post-processing stage of DetectYSF reference/prediction, we additionally introduced a novel “*veracity feature fusion strategy*” based on constructing neighborhood dissemination sub-graph. This strategy involves constructing neighborhood dissemination sub-graph, retrieving news nodes surrounding the current news item under truthfulness prediction, and integrating them through veracity feature transfer.

**Algorithm 1:**
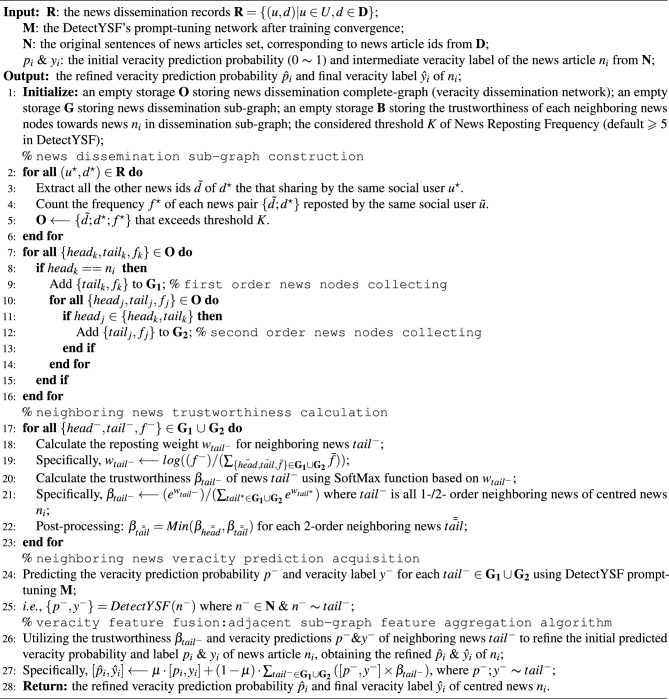
The process of the “*veracity feature fusion strategy*”, towards the news article $n_{i}$.

Specifically, given a set of social context-based news dissemination records $\textbf{R} = \{(u, d) | u \in U, d \in \textbf{D}\}$, where *U* is the user IDs collection involved in reposting news article *d* from $\textbf{D}$, denoting a single user *u*’s engagement in disseminating news article *d* from $\textbf{D}$. Based on the above notations and prerequisites, the “*veracity feature fusion strategy*” by constructing neighborhood dissemination sub-graph can be formulated as follows.

Algorithm 1 intuitively outlines the workflow of the “*veracity feature fusion strategy*”, which is divided into four main steps:

1. Constructing the news dissemination sub-graph: This step involves computing and identifying the neighboring news with first-order and second-order reposting relationships to the current news $n_{i}$ based on the news dissemination records $\textbf{R}$, and then constructing the news dissemination sub-graph (lines 1 to 16 of the algorithm).
2. Calculating the trustworthiness of neighboring news in the sub-graph: In this step, the trustworthiness $\beta _{tail^{-}}$ of all neighboring news nodes within the sub-graph is calculated (lines 17 to 23 of the algorithm).
3. Obtaining initial veracity predictions: Using the DetectYSF prompt-tuning, the initial veracity predictions ${p^{-}, y^{-}}$ of all neighboring news are obtained. These predictions serve as the basis for refining the veracity prediction of the centered news $n_{i}$ (lines 24 to 25 of the algorithm).
4. Veracity feature fusion through adjacent sub-graph feature aggregation algorithm: This step involves fusing the additional veracity-guiding feature biases ${p^{-}, y^{-}}$ into the initial veracity predictions ${p_{i}, y_{i}}$ of the centered news $n_{i}$. This process yields the final refined veracity prediction probability $\hat{p}{i}$ and the final veracity label $\hat{y}{i}$ for news $n_{i}$ (lines 26 to the end of the algorithm). Note that the hyperparameter $\mu$ controls the refining level of news $n_{i}$, ranging from 0 to 1, where larger favors the original prediction and smaller favors the social context-based feature. We chose $\mu = 0.5$ in our DetectYSF, and thus achieved relatively good results.

This “*veracity feature fusion*” post-processing step further refines and optimizes the outcomes of the original prompt-tuning based rumor predictions of DetectYSF, ultimately yielding more accurate performance guided by social contextual knowledge.

## Experiments

To demonstrate the significance of the proposed DetectYSF with previous FEND methods in data-scarce scenarios, this section introduces in detail the experimental procedure, experimental results and corresponding analysis.

### Settings

Several experimental preliminaries, including adopted datasets, state-of-the-art (SOTA) baselines, and hyper-parameters are introduced as follows:

#### Dataset

We assess the performance of our DetectYSF using three commonly utilized real-world benchmark datasets: FakeNewsNet^48^, which includes both the PolitiFact and GossipCop datasets, and the FANG dataset^26^. These datasets feature news articles from trusted fact-checking websites, accompanied by social user engagement data from Twitter, such as repost user IDs. To ensure consistency and reproducibility of results, we followed the same data partitioning method and few-shot setting (with K-shot, where K ranges from 16 to 128) as previous studies. The data were divided into training and testing sets, maintaining an equal ratio of fake news to real news for training purposes.

#### Baseline

To compare our DetectYSF with existing few-shot FEND methods, we evaluated it against several well-known baselines that fall into two categories: “Train-from-Scratch” and “PLM-based” approaches.

The “Train-from-Scratch” baseline methods encompass the following:

1. dEFEND-variant^6^, an adaptation of the hierarchical attention model dEFEND;
2. SentGCN^49^ , which uses Graph Convolutional Network (GCN) methodologies;
3. SAFE’^50^ , a update version of SAFE without the visual component, incorporating a TextCNN module for processing news articles;
4. SentGAT^49^, leveraging Graph Attention Network (GAT) methodologies;
5. GCNFN^11^ , utilizing deep geometric learning to model news dissemination with textual node embedding features;
6. GraphSAGE^26^, which creates a heterogeneous social graph of news articles, sources, and social users for detecting fake news.

The “PLM-based” baseline methods include:

1. BERT-FT^45^, using RoBERTa and a task-specific MLP for predicting news article veracity;
2. PET^11^, which involves task descriptions for PLMs to perform supervised prompt-tuning with cloze questions and verbalizers;
3. RoBERTa-FT^51^, employing BERT and a task-specific MLP for the same purpose;
4. KPT^9^, expanding the label word space with class-related tokens of different granularities and perspectives;
5. Prompt-and-Align^7^, a prompt-tuning method for few-shot FEND that leverages pre-trained LMs and external social context topology knowledge.

To maintain consistency in our experimental setup, we use the official BERT-base model from Huggingface (Huggingface: https://huggingface.co/google-bert/bert-base-uncased [Accessed on 2024.03]). Specifically, the “BertForMaskedLM” model of “bert-base-uncased” serves as the backbone for prompt-tuning in both the baseline methods and our DetectYSF approach.

#### Hyperparameter

We configured the few-shot settings to {16, 32, 64, 128}, with each configuration undergoing three independent training epochs per round. The batch size for each model update was set to 8, and the maximum token sequence length for the DetectYSF’s LM backbone input was constraint at 128. We set the default random seed to 0 and the learning rate for DetectYSF prompt-tuning to 0.00005. The sentence representation-level contrastive learning and sample-level adversarial learning were trained concurrently with LM prompt-tuning, each with a learning rate of 0.000005. For the “sentence representation-level contrastive learning” strategy, the hyperparameter temperature coefficient $\tau$ was set to a default value of 0.05. In the “sample-level adversarial learning” strategy, the weight coefficient $\delta$ controlling the *feature matching* objective loss and the *sample adversarial* loss was set to a default value of 0.5. In the “veracity feature fusion” strategy, the threshold *K* controlling the news reposting frequency in constructing the news dissemination sub-graph was set to 5. Only first-order and second-order neighboring news nodes were considered in the sub-graph feature integration, which were determined to be optimal through experimentation.

#### Metric

To ensure comparability with previous methods, the evaluation metric chosen was *Accuracy*, which measures the ratio of correctly predicted news articles to the total predicted cases, with a higher ratio indicating better performance. *Accuracy* is a standard metric for evaluating general tasks that can be simplified as label classification tasks. It represents the percentage of correctly predicted samples out of all validation/test instances and is defined as Eq. (9):

9 $$\begin{aligned} \text {Accuracy}=\frac{Count_{T}}{Count_{N}} \end{aligned}$$

where $Count_{T}$ denotes the number of correctly predicted samples, and $Count_{N}$ represents the total number of samples. A prediction is considered correct if the predicted news veracity category matches the true label; otherwise, it is considered incorrect.

### Main results

Here, we validate the effectiveness and superiority of our proposed DetectYSF model in the face of few-shot learning scenarios through a series of comparative experiments and quantitative analyses, including comparisons with various state-of-the-art (SOTA) baselines.

#### Baseline comparisons

We evaluated DetectYSF against various baselines for accuracy on the PolitiFact, GossipCop, and FANG datasets, as detailed in Table 1. The baselines were divided into two groups: “Train-from-Scratch” & “PLM-based” as their methodology categories.

**Table 1:** Performance comparisons (% Accuracy) of our DetectYSF and baselines on three benchmarks. Some statistical results are derived from their respective papers^7^. Bold numbers signify the best results, underlined indicate second-best results.

Few-shot FEND approaches	PolitiFact					GossipCop					FANG			
	16	32	64	128		16	32	64	128		16	32	64	128
dEFEND-variant	51.78	54.62	61.06	66.34		50.43	50.35	51.48	52.66		50.19	50.85	50.91	54.60
SentGCN	56.34	51.95	56.81	56.23		49.57	49.33	50.04	53.87		51.35	50.60	52.58	54.45
SAFE’	57.71	54.52	61.91	68.36		51.35	51.20	53.39	57.40		51.90	53.01	55.53	58.35
SetnGAT	56.09	52.99	55.64	58.36		49.54	50.11	50.52	54.65		51.04	51.59	54.09	56.34
GCNFN	55.37	61.86	70.06	84.46		52.18	53.85	62.29	71.89		52.43	56.08	60.12	61.85
GraphSAGE	57.87	60.45	68.72	77.56		51.73	54.10	63.93	69.65		53.32	56.22	58.53	60.44
BERT-FT	61.27	67.48	73.52	77.43		52.45	52.74	55.02	59.28		53.28	54.71	57.02	58.41
PET	64.24	68.09	79.44	80.49		53.69	55.11	59.78	62.97		55.76	56.46	59.13	59.79
RoBERTa-FT	54.80	61.16	78.95	81.44		52.54	54.17	54.09	61.35		51.47	54.56	56.92	60.92
KPT	68.27	70.21	80.40	83.16		53.95	54.70	60.12	62.19		56.87	55.79	60.36	61.38
Prompt-and-Align	85.30	83.73	86.79	89.45		61.15	70.59	74.62	81.60		59.42	60.48	62.37	64.37
DetectYSF	**86.48**	**88.26**	**89.84**	**90.18**		**66.29**	**76.18**	**78.50**	**83.94**		**63.71**	**66.42**	**69.18**	**70.37**

Obviously, the overall results in this table clearly show that DetectYSF outperforms all baselines in terms of accuracy, validating its superior performance. This underscores the importance of integrating the “*sample-level adversarial learning*” strategy, the *veracity feature fusion strategy*” based on neighborhood dissemination sub-graph, and the *news veracity dissemination consistency*” theory into the original LM-based FEND approach.

Additionally, it is apparent that the “Train-from-Scratch” methods in first group, which utilize task-specific neural architectures, rely heavily on the size and quality of the supervised data. For example, GCNFN’s performance on the PolitiFact dataset is 55.37% with 16-shot, compared to 84.46% with 128-shot, indicating a substantial increase of approximately 29.09% in accuracy. This highlights the limitations of supervised learning due to its dependency on large, high-quality labeled datasets. In real-world scenarios, acquiring such datasets is often costly, time-consuming, and labor-intensive. Moreover, labeled data may be sparse or imbalanced, especially in niche domains or emerging areas where new data is continuously generated. Thus, techniques such as semi-supervised learning, unsupervised learning, and leveraging pre-trained models (PLMs) become essential, reducing the dependency on extensive labeled datasets and enhancing learning efficiency and performance.

For the FANG dataset, the detection of news authenticity involves the relationships among three distinct node categories: users, news articles, and media sources. Unlike datasets like PolitiFact and GossipCop, the reliance on semantic information from news content is less significant. When introduced with FANG, even approaches like GraphSAGE demonstrated limited improvements, achieving only a 7.12% increase in accuracy from 53.32% at 16-shot to 60.44% at 128-shot. This modest gain is reflective of other models as well; for instance, KPT and Prompt-and-Align only reached 61.38% and 64.37% accuracy, respectively, at 128-shot. Despite the expectations from traditional “Train-from-Scratch” and “PLM-based” approaches, they did not perform as well in few-shot learning scenarios. On the contrary, our method, which incorporates the construction of the social context veracity dissemination consistency network along with neighborhood dissemination sub-graph and dissemination consistency-guided strategies, achieved results that significantly exceeded the performance of traditional PLM-based methods on the FANG dataset. Specifically, our method achieved 63.71% accuracy in 16-shot (an approximate 4% absolute improvement), 66.62% accuracy in 32-shot (about a 6% absolute improvement), 69.18% accuracy in 64-shot (around a 7% absolute improvement), and 70.37% accuracy in 128-shot (about a 6% absolute improvement) compared to Prompt-and-Align. This marks a significant advancement with practical implications.

Moreover, the statistical analysis shows that “PLM-based” methods significantly outperform “Train-from-Scratch” methods, highlighting the advantage of using rich pre-trained knowledge in PLMs to address label scarcity. These methods are better suited for few-shot FEND tasks compared to developing task-specific neural architectures for fake news detection. Additionally, the comparison between Prompt-And-Align, KPT, and PET methods against simple fine-tuning on BERT/RoBERTa indicates that the prompt learning paradigm more effectively harnesses the prior linguistic knowledge learned during PLM pre-training, resulting in better performance than the PLM fine-tuning paradigm.

In conclusion, the baseline comparisons conclusively demonstrate that the self-supervised contrastive learning, the semi-supervised adversarial learning, the “news veracity dissemination consistency” theory provides crucial insights for few-shot fake news detection. Especially, the “*veracity feature fusion*” post-processing step acts as essential external knowledge for few-shot fake news detection tasks, enhancing the original model’s FEND capability to predict rumors accurately. Once again, our DetectYSF can be regarded as the state-of-the-art (SOTA) solution for rumor detection in low-resource scenarios.

### Ablated studies

Here, we focus on the DetectYSF framework itself by conducting a series of ablation experiments involving modifications, removals, and reconfigurations. These experiments aim to explore the effectiveness of the inherent characteristics of the DetectYSF model and assess the necessity of its primary components and modules.

#### Effectiveness of DetectYSF’s primary components

As presented in Table 2. The effectiveness and superiority of DetectYSF’s primary components—including the *sentence representation contrastive learning strategy*, the *sample-level adversarial learning strategy*, and the *neighborhood dissemination sub-graph veracity feature fusion*—are demonstrated through ablation studies that respectively remove their core components, specifically in the DetectYSF Model Variants group.

Firstly, the *sentence representation contrastive learning strategy* aims to enhance the robustness and convergence of the model by increasing the similarity of positive pairs while decreasing the similarity of negative pairs. For this component, comparing the results of the “REMOVE-contrastive learning” group with the default DetectYSF, we can observe that removing this component results in performance drops across all datasets and shot settings. Specifically, for PolitiFact with 16, 32, and 64 shots, the accuracy decreases by 3.56%, 4.05%, and 2.11%, respectively. Similarly, significant declines are observed for the GossipCop and FANG datasets, indicating the crucial role of this strategy in maintaining high accuracy. For instance, in the FANG dataset, the accuracy drops by 5.18% for 16-shot, 4.57% for 32-shot, and 6.72% for 64-shot settings, highlighting its contribution to the model’s performance in data-scarce scenarios.

Then, the *sample-level adversarial learning strategy* aims to improve the generalization and robustness of the model by utilizing adversarial examples. Comparing the ablation results of the “REMOVE-adversarial learning” group with the default setup reveals noticeable performance degradation when this component is removed. Specifically, for the PolitiFact dataset with 16, 32, and 64 shots, accuracies decrease by 3.67%, 1.79%, and 3.73%, respectively. The impact is even more pronounced in the GossipCop and FANG datasets, with drops of 4.10% and 6.53% for GossipCop (16 and 32 shots), and 3.04% and 1.5% for FANG (16 and 32 shots). These phenomenons underscore the importance of adversarial learning in enhancing the DetectYSF’s capability to handle noisy and deceptive inputs.

It is worth noting the significant difference in the impact of removing the *contrastive learning strategy* compared to the *adversarial learning strategy*. When the contrastive learning component is removed, DetectYSF experiences more substantial performance decreases across datasets and shot settings than when adversarial learning is removed. For example, the removal of contrastive learning results in accuracy drops of 1.56%, 3.05%, and 2.11% for FANG with 16, 32, and 64 shots, respectively. In contrast, removing adversarial learning results in smaller drops, such as 3.04%, 1.5%, and 1.84% for FANG. These findings indicate that the *contrastive learning strategy* plays a more crucial role in enhancing DetectYSF’s performance in few-shot scenarios. By effectively distinguishing between similar and dissimilar pairs, contrastive learning significantly bolsters the model’s robustness and convergence, offering greater benefits in data-scarce conditions, making it more essential for DetectYSF.

**Table 2:** The performance comparison statistics of DetectYSF’s variants which remove specific components (numbers underline are default).

DataSets (K-shot)		PolitiFact			GossipCop			FANG		
		16	32	64	16	32	64	16	32	64
DetectYSF model variants	*REMOVE*-contrastive learning	82.92 $\downarrow$ 3.56%	84.21 $\downarrow$ 4.05%	87.73 $\downarrow$ 2.11%	62.24 $\downarrow$ 2.05%	67.47 $\downarrow$ 8.71%	69.17 $\downarrow$ 9.33%	58.53 $\downarrow$ 5.18%	61.85 $\downarrow$ 4.57%	62.46 $\downarrow$ 6.72%
	*REMOVE*- adversarial learning	84.81 $\downarrow$ 1.67%	86.47 $\downarrow$ 1.79%	86.11 $\downarrow$ 3.73%	62.19 $\downarrow$ 4.10%	69.65 $\downarrow$ 6.53%	74.62 $\downarrow$ 3.88%	60.67 $\downarrow$ 3.04%	64.92 $\downarrow$ 1.5%	67.34 $\downarrow$ 1.84%
	*REMOVE*- veracity feature fusion	77.37 $\downarrow$ 9.11%	82.53 $\downarrow$ 5.73%	84.51 $\downarrow$ 5.33%	59.28 $\downarrow$ 7.01%	63.45 $\downarrow$ 12.73%	65.29 $\downarrow$ 13.21%	55.42 $\downarrow$ 8.29%	60.42 $\downarrow$ 6.00%	62.93 $\downarrow$ 6.25%
DetectYSF (default)		86.48	88.26	89.84	66.29	76.18	78.50	63.71	66.42	69.18

Lastly, the *neighborhood dissemination sub-graph veracity feature fusion* strategy leverages veracity features from neighboring news nodes to enhance the DetectYSF’s predictions. The ablation results of “REMOVE - veracity feature fusion” group reveal that removing this component results in the most significant performance declines across all datasets, especially in the GossipCop and FANG datasets. Specifically, for the GossipCop dataset with 16, 32, and 64 shots, accuracies drop by 7.01%, 12.73%, and 13.21%, respectively. Similarly, for the FANG dataset, the decreases are 8.29%, 6.00%, and 6.25% across the same shot settings. This substantial impact highlights the effectiveness of incorporating social context to refine and improve veracity predictions, making it a critical component of the DetectYSF framework.

Overall, this ablation studies clearly demonstrate the vital contributions of each component to the overall effectiveness of DetectYSF. By synergizing these strategies, DetectYSF achieves superior performance in few-shot fake news detection, outperforming state-of-the-art baselines and proving its robustness and generalizability in low-resource scenarios.

#### Ablations of sentence-level contrastive learning

Here, we primarily focus on the implementation details of the “sentence-level contrastive learning” strategy, demonstrating the rationality and effectiveness of this design through a series of ablation experiments.

1. Effectiveness of unsupervised/supervised contrastive learning. As shown in Table 3, we analyze the importance of the *unsupervised contrastive learning* and *supervised contrastive learning* methodologies within the Sentence Representation Contrastive Learning Strategy in DetectYSF.

**Table 3:** The performance comparisons between the revised DetectYSF, which omits unsupervised-/supervised- contrastive learning, and the initial DetectYSF implementation. (Numbers underline are default.).

DataSets (K-shot)		PolitiFact				GossipCop				FANG			
		16	32	64	128	16	32	64	128	16	32	64	128
DetectYSF model variants	Unsupervised removed	83.49 $\downarrow$ 2.99%	88.93 $\uparrow$ 0.67%	88.92 $\downarrow$ 0.92%	89.07 $\downarrow$ 1.11%	65.64 $\downarrow$ 0.65%	74.31 $\downarrow$ 1.87%	73.81 $\downarrow$ 4.69%	81.74 $\downarrow$ 2.20%	59.59 $\downarrow$ 4.12%	63.48 $\downarrow$ 2.94%	67.57 $\downarrow$ 1.61%	69.92 $\downarrow$ 0.45%
	supervised removed	85.37 $\downarrow$ 1.11%	87.91 $\downarrow$ 0.35%	88.39 $\downarrow$ 1.45%	89.73 $\downarrow$ 0.45%	63.38 $\downarrow$ 2.91%	72.95 $\downarrow$ 3.23%	75.95 $\downarrow$ 2.55%	81.51 $\downarrow$ 2.43%	58.41 $\downarrow$ 5.30%	65.26 $\downarrow$ 1.16%	65.38 $\downarrow$ 3.80%	71.31 $\uparrow$ 0.94%
DetectYSF (default)		86.48	88.26	89.84	90.18	66.29	76.18	78.50	83.94	63.71	66.42	69.18	70.37

Firstly, the importance of *unsupervised contrastive learning* strategy is evident from the performance degradation observed upon its removal. For PolitiFact, the removal resulted in a performance drop across most K-shot settings, with the 16-shot accuracy decreasing by 2.99% and the 64-shot accuracy decreasing by 0.92%. Similarly, for the GossipCop dataset, significant accuracy drops were noted, especially at higher K-shot settings, such as a 4.69% decrease in 64-shot accuracy and a 2.20% decrease in 128-shot accuracy. In the FANG dataset, although the impact was less consistent, there was still a notable drop of 4.12% at 16-shot and 2.94% at 32-shot. These underscore the critical role of *unsupervised contrastive learning* in maintaining high model performance.

Furthermore, the importance of *supervised contrastive learning* strategy is demonstrated by the performance degradation observed upon its removal. For PolitiFact, removing this strategy led to a smaller yet noticeable performance drop, with the 16-shot accuracy decreasing by 1.11% and the 64-shot accuracy decreasing by 1.45%. In the GossipCop dataset, the 64-shot setting showed a significant drop of 2.55%, while the 128-shot setting showed a decline of 2.43%. In contrast, the FANG dataset experienced less severe effects from its removal, with an improvement observed in the 128-shot setting (+ 0.94%), indicating that *supervised contrastive learning* might be less critical in some high-shot settings. These results highlight the role of *supervised contrastive learning* in enhancing model performance, though its importance may vary depending on the dataset and the amount of available data.

Overall, both the two methodologies are crucial for enhancing the performance of DetectYSF, highlighting the importance of incorporating both unsupervised and supervised contrastive learning strategies to achieve optimal few-shot FEND performance in DetectYSF. *Unsupervised contrastive learning* is vital for improving the generalizability and robustness of sentence representations in low-resource scenarios. Meanwhile, *supervised contrastive learning* also contributes to performance improvements, but its impact might be more context-dependent, varying with the dataset and the amount of available data.

2. Influence of different similarity distance metrics. As provided in Table 4, we evaluate the influence of different similarity distance metrics that optimizes *un-/supervised contrastive learning objective* on the DetectYSF detection performance in few-shot FEND, acrossing various K-shot settings (16, 32, and 64 shots) and three datasets: PolitiFact, GossipCop, and FANG. The distance metrics assessed are L1-Distance, L2-Distance, and Cosine Similarity, which are introduced as follows:

**Table 4:** The performance comparisons of DetectYSF variants which adopt different similarity distance metrics in sentence-level contrastive learning (numbers underline are default).

DataSets (K-shot)		PolitiFact			GossipCop			FANG		
		16	32	64	16	32	64	16	32	64
DetectYSF-variants	L1-Distance Margin	84.13 $\downarrow$ 2.35%	87.34 $\downarrow$ 0.92%	86.72 $\downarrow$ 3.12%	63.84 $\downarrow$ 2.45%	75.13 $\downarrow$ 1.05%	75.95 $\downarrow$ 2.55%	61.44 $\downarrow$ 2.27%	64.92 $\downarrow$ 1.50%	67.73 $\downarrow$ 1.45%
	L2-Distance Margin	84.13 $\downarrow$ 2.35%	86.21 $\downarrow$ 2.05%	87.97 $\downarrow$ 1.87%	67.79 $\uparrow$ 0.87%	74.31 $\downarrow$ 1.13%	77.46 $\downarrow$ 1.04%	64.41 $\uparrow$ 1.30%	65.29 $\downarrow$ 1.13%	66.81 $\downarrow$ 2.37%
DetectYSF (default)	Cosine Similarity	86.48	88.26	89.84	66.29	76.18	78.50	63.71	66.42	69.18

- L1-Distance (Manhattan Distance): This metric sums the absolute differences across dimensions. While it can be useful in some contexts, its performance in high-dimensional spaces (like those used in NLP tasks) can be less effective due to the “curse of dimensionality,” leading to reduced discriminative power in few-shot learning.
- L2-Distance (Euclidean Distance): This metric calculates the straight-line distance between two points in space. Similar to L1-Distance, its effectiveness diminishes in high-dimensional spaces, making it less suitable for tasks requiring nuanced feature differentiation, such as few-shot fake news detection.
- Cosine Similarity: This metric measures the cosine of the angle between two vectors, effectively capturing the directional alignment between feature representations. Its effectiveness in few-shot learning tasks can be attributed to its ability to focus on the relative orientation of vectors rather than their magnitude, which is crucial when dealing with sparse data.

The comparison of L1-Distance and L2-Distance metrics with Cosine Similarity reveals that both L1 and L2 distances generally underperform across all datasets and K-shot settings. The margins, indicating changes in accuracy, are consistently negative for both L1 and L2 distances, suggesting a drop in performance when these metrics are used instead of Cosine Similarity. These performance drops are particularly noticeable in lower K-shot settings (16 and 32 shots) for the GossipCop and FANG datasets. In contrast, Cosine Similarity consistently yields the highest accuracy across all datasets and K-shot settings. Specifically, for PolitiFact, the performance gap between Cosine Similarity and L1/L2 distances narrows as the number of shots increases (from 16 to 64 shots), yet Cosine Similarity maintains a clear advantage. Similar trends are observed for GossipCop and FANG, the margin improvements show that Cosine Similarity is particularly beneficial, with more substantial performance drops for L1 and L2 distances, further reaffirming the effectiveness of Cosine Similarity.

Overall, it clearly indicates that Cosine Similarity is the superior distance metric for optimizing *un-/supervised contrastive learning objective* and thus enhancing the generalization and detection abilities of DetectYSF in few-shot FEND tasks. It consistently outperforms L1-/L2- distances across different datasets and K-shot settings, demonstrating its robustness and reliability in low-resource scenarios.

#### Ablations of sample-level adversarial learning

In this context, we concentrate on the specific design of the “sample-level adversarial learning” strategy, demonstrating its rationality and superiority through a series of ablation experiments.

1. Superiority of Noise-MLP Engine & NegSeq-LMEncoder Engine combination. We evaluate the performance impact by removing different components of the *sample-level adversarial learning* strategy from DetectYSF, providing the ablated results in Table 5. The first group completely removes *sample-level adversarial learning*. The second and third groups individually remove one generator each, specifically the Noise-MLP Engine and the NegSeq-LMEncoder Engine. The last group presents the complete performance results of DetectYSF. Noise-MLP Engine uses a Multilayer Perceptron (MLP) driven by random noise to generate adversarial examples, adding diversity and robustness to the model, while NegSeq-LMEncoder Engine focuses on generating adversarial examples using LM encoder and negative sequences.

**Table 5:** The comparison results evaluating the performance impact by removing different components of the *sample-level adversarial learning* strategy from DetectYSF. The best is highlighted in bold, the second-best is underlined, and the worst is italicized.

DataSets (K-shot)			PolitiFact			GossipCop			FANG		
			16	32	64	16	32	64	16	32	64
DetectYSF-variants	REMOVE-contrastive learning		*82.92*	*84.21*	87.73	*62.24*	*67.47*	*69.17*	*58.53*	*61.85*	*62.46*
	PART-contrastive learning	+ Noise-MLP engine	84.93	85.34	*86.47*	64.92	70.73	*69.17*	59.98	63.45	65.34
		+ NegSeq-LMEncoder Engines	**87.19**	87.19	88.92	66.01	73.20	75.56	**63.71**	65.19	67.67
DetectYSF (default)			86.48	**88.26**	**89.84**	**66.29**	**76.18**	**78.50**	**63.71**	**66.42**	**69.18**

As seen in Table 5, removing the contrastive learning strategy significantly decreases the DetectYSF performance. For example, in the 16-shot setting for PolitiFact, accuracy drops from 86.48 to 82.92%, and in the 16-shot setting for GossipCop, accuracy drops from 66.29 to 62.24%. These results indicate that the contrastive learning strategy is crucial for maintaining high performance. Meanwhile, the default DetectYSF configuration, which includes both engines, consistently achieves higher accuracy across all datasets and K-shot settings compared to variants where one or both engines are removed. In summary, the Noise-MLP Engine and NegSeq-LMEncoder Engine combination significantly boosts the robustness and generalization abilities of DetectYSF. Their removal leads to substantial performance drops, underscoring their critical roles in the *sample-level adversarial learning* strategy of DetectYSF.

Moreover, comparing the results of different GAN’s generator removals, it is evident that NegSeq-LMEncoder Engine provides more significant performance improvements and plays a more critical role than Noise-MLP Engine. For instance, in the 16-shot setting on PolitiFact, removing the NegSeq-LMEncoder Engine results in an accuracy of 84.93%, whereas removing the Noise-MLP Engine results in an accuracy of 87.19%. This phenomenon is also observed in other datasets, such as the 64-shot setting for GossipCop, where accuracy is 69.17% when the NegSeq-LMEncoder Engine is removed, compared to 75.56% when the Noise-MLP Engine is removed. Particularly in the 16-shot settings on the PolitiFact and FANG datasets, the performance of the NegSeq-LMEncoder Engine is comparable to that of the complete DetectYSF. For example, in the 16-shot setting on the PolitiFact dataset, the accuracy of the complete DetectYSF is 86.48%, while the accuracy is 87.19% when only the Noise-MLP Engine is removed, showing almost no difference. Similarly, in the 16-shot setting on the FANG dataset, the accuracy of the complete DetectYSF is 63.71%, and the accuracy is also 63.71% when only the Noise-MLP Engine is removed.

These findings indicate that NegSeq-LMEncoder Engine has a more significant impact on optimizing sentence semantic representation and plays a critical role in DetectYSF. It effectively improves the robustness and generalization ability of DetectYSF by generating more diverse and high-quality adversarial samples than Noise-MLP Engine, playing a vital role in enhancing DetectYSF in few-shot FEND tasks.

2. Efficiency of “feature matching” objective. Here, we analyze the effectiveness of the “feature matching” objective by comparing the performance of DetectYSF with and without the “feature matching” objective across various K-shot settings and multiple datasets.

**Figure 3:**
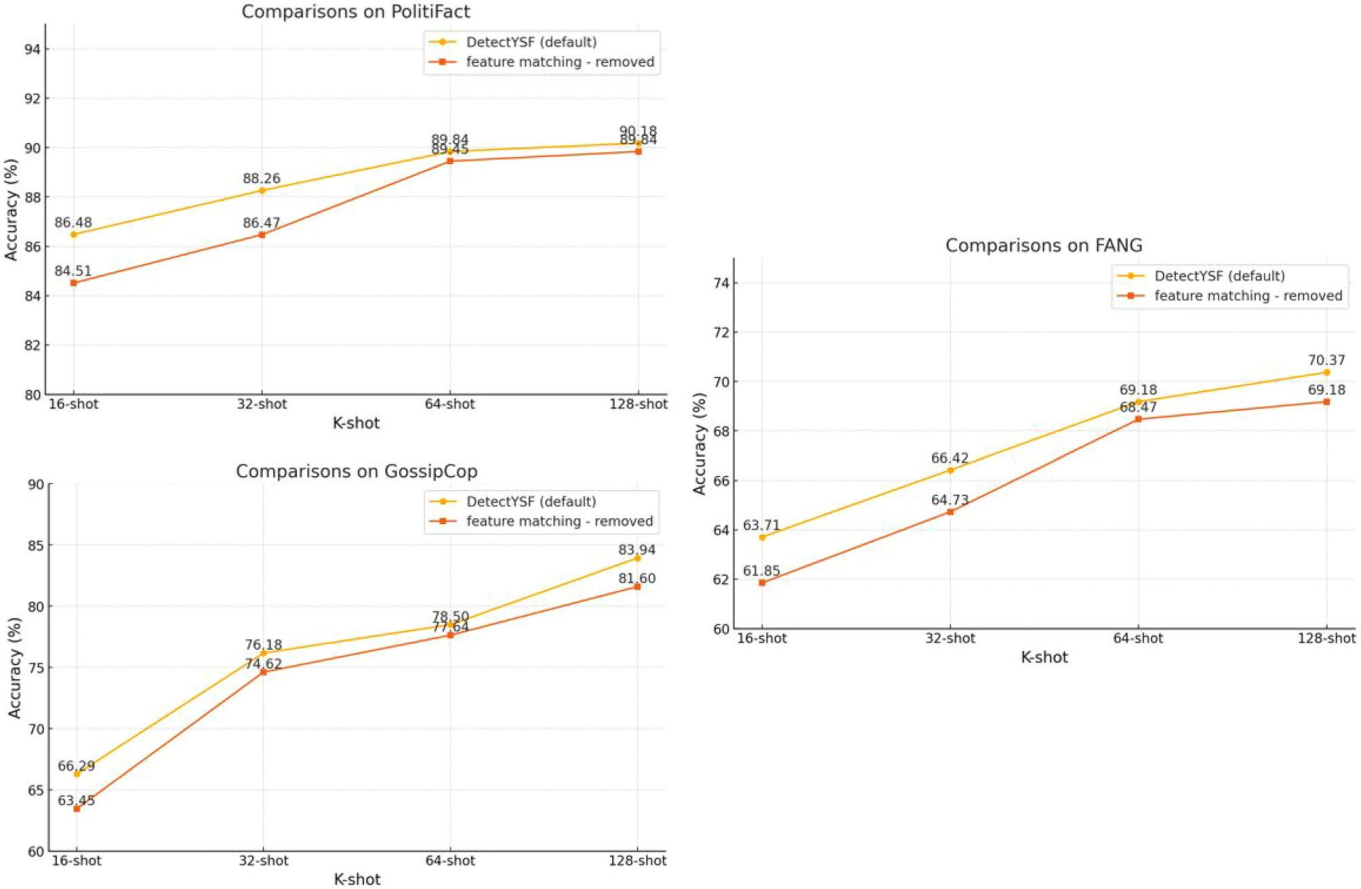
Comparative Performance of DetectYSF with and without “feature matching” objective on PolitiFact, GossipCop, and FANG datasets and various few-shot settings.

As depicted in Fig. 3, we present the accuracy comparison of DetectYSF and its variants in different K-shot settings (16-, 32-, 64-, and 128-shot) on the PolitiFact, GossipCop, and FANG datasets. Figure 3 compares the default DetectYSF, which includes the “feature matching” objective in the adversarial learning optimization process, against a variant where the feature matching objective is removed. For PolitiFact, the default DetectYSF consistently outperforms the variant without feature matching across all K-shot settings, with the most notable improvements in lower K-shot settings. Similar trends are observed in GossipCop and FANG. The above observations illustrate the effectiveness of the “feature matching” objective in improving few-shot performance, underscore the critical role of the feature matching objective in the adversarial learning of DetectYSF.

#### Ablations of neighborhood sub-graph veracity feature fusion

1. Effect of trustworthiness $\beta$ in adjacent feature aggregation

In the “veracity feature fusion” strategy, we calculate the trustworthiness $\beta _{tail^{-}}$ of each news node in an adjacent sub-graph. This trustworthiness value is then used for adaptive weighted feature fusion. The specific formula is given by: $[\hat{p}_{i}, \hat{y}_{i}] \longleftarrow \mu \cdot [p_{i}, y_{i}] + (1-\mu )\cdot \sum _{tail^{-} \in \mathbf {G_{1}} \cup \mathbf {G_{2}}}\left( \left[ p^{-} , y^{-}\right] \times \beta _{tail^{-}}\right)$. We conducted comparative experiments on this veracity feature fusion strategy, which considers the strength of news reposting relationships in the sub-graph. Specifically, we modified the feature integration algorithm of the neighborhood sub-graph veracity feature fusion to an averaged weighted veracity feature fusion strategy. The updated formula is: $[\hat{p}_{i}, \hat{y}_{i}] \longleftarrow \mu \cdot [p_{i}, y_{i}] + (1-\mu )\cdot \sum _{tail^{-} \in \mathbf {G_{1}} \cup \mathbf {G_{2}}}\left( \left[ p^{-} , y^{-}\right] \times \frac{1}{|\mathbf {G_{1}}| \cup |\mathbf {G_{2}}|}\right)$. Here, $\frac{1}{|\mathbf {G_{1}}| \cup |\mathbf {G_{2}}|}$ ensures that the features of adjacent news nodes are evenly distributed to the central news node. We performed comparative experiments under 16-shot, 32-shot, and 64-shot settings across the three datasets. And the statistical results are shown in Table 6.

**Table 6:** The performance comparisons of DetectYSF variants that use trustworthiness driven adaptive weighted feature fusion with averaged veracity feature fusion.

DataSets		PolitiFact			GossipCop			FANG		
(K-shot)		16	32	64	16	32	64	16	32	64
DetectYSF (variants)	REMOVE-veracity fusion	77.37	82.53	84.51	59.28	63.45	65.29	55.42	60.42	62.93
	Averaged fusion	83.62	85.91	86.07	62.24	69.18	75.19	58.71	63.14	67.22
DetectYSF (default)	Adaptive weighted	**86.48**	**88.26**	**89.84**	**66.29**	**76.18**	**78.50**	**63.71**	**66.42**	**69.18**

In this table, DetectYSF (variants)-REMOVE *veracity fusion* shows the performance when the veracity fusion component is removed; DetectYSF (variants)-*averaged fusion* displays results when using an averaged weighted veracity feature fusion strategy; DetectYSF (default)-*adaptive weighted* presents the results for the default DetectYSF model, which employs the trustworthiness-driven adaptive weighted feature fusion strategy. The table show that for PolitiFact, the REMOVE veracity fusion variant performs the lowest, peaking at 84.51% for 64-shot, while the averaged fusion improves to 86.07%, and the default adaptive weighted fusion achieves the best results at 89.84%. In GossipCop, REMOVE veracity fusion again scores the lowest, with a maximum of 65.29% for 64-shot. The averaged fusion shows better results at 69.18% for 32-shot, while the default adaptive weighted fusion reaches 78.50% for 64-shot. Similar trends are observed in FANG.

These results clearly demonstrate that the default DetectYSF with adaptive weighted veracity feature fusion outperforms the variants with both removed and averaged fusion strategies across all datasets and shot settings, highlighting the effectiveness of incorporating trustworthiness-driven adaptive weighted feature fusion in few-shot FEND scenarios. This further validates the “*news veracity dissemination consistency*” theory, which posits that the more frequently a set of news articles is shared by the same social users, the higher the consistency of their veracity characteristics.

2. Optimal refining level of sub-graph feature aggregation. The hyperparameter $\mu$ in the “*veracity feature fusion strategy*” is used to control the extent of feature integration between the veracity features of neighboring news nodes and the original centered news veracity prediction, ranging from 0 to 1. By experimenting with different values of $\mu$, we aim to determine the optimal value that achieves the best rumor detection performance.

**Figure 4:**
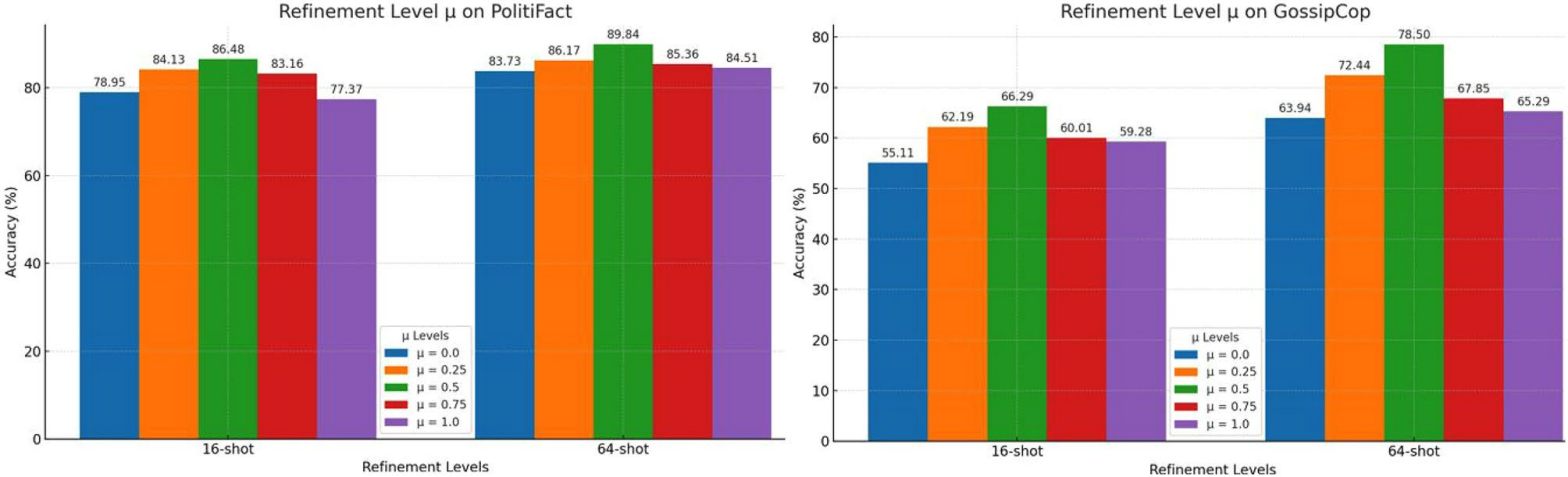
The performance comparisons of the impacts on DetectYSF variants towards different hyperparameter $\mu$ configurations.

Specifically, in the experiments, we conducted comprehensive ablated experiments on various low-resource scenarios, including range from PolitiFact to GossipCop using 16-shot and 64-shot settings. As shown in Fig. 4, we conducted a grid search for the hyperparameter $\mu$, setting it to five different refinement levels ($\mu$ = 0.0, $\mu$ = 0.25, $\mu$ = 0.5, $\mu$ = 0.75, $\mu$ = 1.0). Based on these experimental results, we clearly observe that when $\mu$ is around 0.5, DetectYSF achieves optimal predictive performance. In contrast, when $\mu$ approaches the extremes, closing to 0 or 1, the rumor prediction performance of DetectYSF significantly declines. This confirms that properly tuning the refinement level $\mu$ is a critical step in the effective modeling of DetectYSF.

## Conclusion and perspective

In this work, we introduced a novel framework named Detection Yet See Few (DetectYSF) for few-shot fake news detection, designed to operate effectively in low-resource scenarios. DetectYSF leverages the strengths of contrastive learning and adversarial learning, combining semi-supervised learning and self-supervised learning to enhance the robustness and generalizability of Transformer-based pre-trained LMs. By adopting a Masked LM-based pseudo prompt learning paradigm for model tuning, DetectYSF achieves superior performance with limited supervised data. DetectYSF also integrates a unique strategy based on the “news veracity dissemination consistency” theory, utilizing social context clues to further refine and optimize detection accuracy. Our experimental results demonstrate that DetectYSF surpasses many state-of-the-art (SOTA) baselines under various few-shot settings in terms of detection accuracy across three widely-used benchmarks. For instance, DetectYSF achieved an accuracy of 83.94% on GossipCop dataset, compared to 62.97% by PET and 62.19% by KPT. The promising performance of DetectYSF underscores its potential as a powerful tool for automatic rumor detection in data-scarce scenarios, contributing significantly to mitigating the spread of misinformation in various real-world applications. Our framework and findings provide a foundation for future research in few-shot fake news detection, aiming to sustain the health and integrity of information dissemination within society.

In future work, we plan to further enhance DetectYSF by exploring several directions. First, we will refine the integration of social context clues by incorporating more sophisticated network analysis techniques and richer social media data, which can provide a deeper understanding of the dissemination patterns and improve DetectYSF’s fake news detection ability. Second, we will investigate the application of DetectYSF in multilingual and cross-lingual settings to address the global nature of misinformation, making it more versatile and applicable to a wider range of contexts. Third, we plan to experiment with more diverse datasets for pre-training, and more advanced semi-/self-supervised learning techniques to further improve DetectYSF in low-resource environments. Moreover, we also see potential in combining DetectYSF with other modalities, such as images and videos, to create a multi-modal fake news detection system. Finally, we will enhance the scalability and efficiency of DetectYSF to ensure it can be deployed in real-time applications. By addressing these aspects, we aim to develop a more robust, versatile, and efficient fake news detection system that can better serve society.

## Data Availability

The used datasets will be made available upon request to the email of Weiqiang Jin.

## Code availability

Full code to replicate the experiments are available from Github: https://github.com/albert-jin/Self-Supervi-sed-fewshot-FEND-Enhance.
